# Sex specific effects of adoptive Tregs transfer on the brain and periphery in maternal immune activation offspring rescuing immune dysregulation

**DOI:** 10.21203/rs.3.rs-8414528/v1

**Published:** 2026-02-01

**Authors:** Destanie Rose, Rachel Moreno, Hadley Osman, Megan Rowland, Jill Silverman, Annie Ciernia, Paul Ashwood

**Affiliations:** University of California, Davis; University of California, Davis; University of California, Davis; University of British Columbia; University of California, Davis; University of British Columbia; University of California, Davis

**Keywords:** maternal immune activation (MIA), autism, schizophrenia, immune regulation, regulatory T cells (Tregs), inflammation, behavior, TH17 cells, sex, gene expression

## Abstract

Autism spectrum disorder (ASD) is characterized by atypical communication, social interactions, and restricted interests. In ASD, there are dysfunctional immune regulatory control mechanisms that can lead to immune activation. Notably lower frequencies of regulatory T cells (Tregs) and reduced immunosuppressive cytokines are reported and associated with more impaired behaviors impacting both individuals with autism and their families. Therefore, therapeutic approaches that enhance immune regulation may offer substantial benefits. Using the maternal immune activation (MIA) model, we investigated whether adoptive transfer of wildtype Tregs into MIA offspring recipients could rescue immune activation, brain transcriptome changes and behaviors exhibited in adult MIA offspring. We also aimed to explore potential sex-differences in responses. In male but not female MIA offspring, Tregs transfer reduced the frequency of T helper (T_H_)-17 (RORγT^+^ CD4^+^) T cells in both the mesenteric lymph node (MLN) and spleen. Moreover, the frequency of CD25^+^Foxp3^+^ T cells was increased in the MLN and spleen of male but not female MIA offspring following Treg transfer. Splenocytes from male MIA offspring receiving Tregs showed reduced production of inflammatory cytokines (e.g., IL-6 and TNFα) following PMA/Ionomycin stimulations. In contrast, female MIA offspring that received Tregs exhibited different cytokine profiles characterized by increased production of cytokines, including GM-CSF, IFNγ, and IL-10. In the brain, bulk mRNA sequencing in the cerebellum, frontal cortex, and hippocampus revealed that Tregs-treated male MIA offspring had differentially expressed genes involved in neurodevelopmental disorders, synaptic function, and epigenetic regulation. Minimal gene expression differences were observed in female counterparts. There was significant improvement in self-grooming behaviors in males MIA offspring that received Tregs. In females, social novelty improved after Tregs treatment. In summary, adoptive Tregs transfer reduced systemic inflammation, brain transcription and behavior alterations in a sex dependent manner in the context of MIA. These findings suggest that adoptive Treg transfer may represent a viable therapeutic avenue for mitigating systemic inflammation and comorbidities associated with MIA and neurodevelopmental disorders such as ASD.

## Introduction

Neurodevelopmental disorders (NDD) are becoming increasingly prevalent. Recent population-based data suggested that autism spectrum disorder (ASD), an NDD defined by restricted interests and atypical social interactions, is now diagnosed in 1 in 31 children, compared to 1 in 150 children just two decades ago [1]. The causes behind this increase remain unclear, though it is widely accepted that both genetic and environmental factors contribute to ASD etiology. Despite the heterogeneity introduced by individual genetics and unique environmental exposures, many risk factors converge on immune dysregulation. Evidence from epidemiology studies demonstrates that maternal immune activation (MIA) during pregnancy, from infections, asthma or autoimmune conditions, is associated with an increased risk of ASD in children [2-4]. Moreover, offspring of animal models of MIA exhibit many NDD traits [2, 5, 6]. An increase in maternal inflammatory cytokines during gestation including interleukin (IL)-6 and IL-17 are thought to disrupt neurodevelopment in the fetus by binding to receptors on fetal neurons and affecting neural circuitry and synapse connectivity [7, 8]. Long-term immune activation in the periphery has also been shown, including increased numbers of T helper (T_H_17) cells and innate immune cell activation [9-12].

Children with ASD frequently exhibit chronic immune abnormalities, characterized by heightened immune activation and impaired regulatory mechanisms [13]. This inflammatory profile includes increased innate and adaptive immune cells with pro-inflammatory phenotypes [14]. Importantly, inflammation in ASD is not restricted to a single tissue. Elevated levels of pro-inflammatory cytokines have been observed in the brain, blood, and gastrointestinal (GI) tract [15-18]. A likely contributor to this dysregulated immune state is a reduction in immunosuppressive mechanisms, particularly regulatory T cells (Tregs). Tregs are essential components of the adaptive immune system that, if ablated, are associated with severe autoimmunity and cognitive impairment in both humans and animal models [19]. Tregs suppress immune activation through two broad categories of mechanisms: secretion of soluble factors and cell-contact–dependent regulation. Soluble mechanisms include the release of anti-inflammatory cytokines such as IL-10, IL-35, and transforming growth factor-beta-1(TGF-β1). In individuals with ASD, the concentrations of these cytokines are decreased, and lower levels are associated with more severe behavioral symptoms and increased comorbidities [20-22]. While the soluble arm of Tregs function has been relatively well studied, the extent of impairment in contact-dependent mechanisms remains largely unknown. However, given that in ASD there is reduced expression of the transcription factor Foxp3, that controls the expression of contact-dependent receptors such as CTLA-4, it is likely that these mechanisms are also compromised [23, 24].

Therapies that directly target Tregs function in ASD remain limited. Clinical trials with corticosteroids (Prednisolone) and immune-suppressive agents (Celecoxib) that inhibit pro-inflammatory cytokine production and blunt effector T lymphocyte activation, have shown improvement of core-ASD and associated behaviors, including social interactions, language, vocalization, stereotypic behavior, irritability and emotional regulation [25-29]; however, these trials were often small. One study evaluating a toxoplasma-induced variation of the MIA model found that the transfer of pathogen-specific maternal Tregs led to improved behaviors in offspring [30]. A more recent study evaluating the effects of low-dose IL-2, meant to promote Tregs expansion, in BTBR mice found reduced neuroinflammation, improved behavior, and increased frequencies of Tregs in the periphery [31]. Other preliminary studies indicate that modulating Tregs activity may ameliorate chronic inflammation and, in turn, improve behavior and inflammation [32]. For instance, in ASD, umbilical cord blood transfer that may alter immune cell phenotypes has also been shown to improve some behaviors [33-35]. In this study, we investigated the effects of adoptive Tregs transfer from syngeneic wildtype donor mice into offspring from the MIA treated dams. Our findings revealed sex-specific differences following Tregs transfer on immune cell composition, immune activation, brain transcriptomics and behavior, that were more pronounced in male MIA offspring than in females.

## Materials & Methods

### Mice.

C57Bl/6N (C57) mice were obtained from Taconic Farms (Rensselaer, NY). Mice were maintained by the campus Teaching and Research Animal Care Services (TRACS), at University of California, Davis at ambient room temperature on a 12-hour light/dark cycle with water and food available *ad libitum*. All procedures were approved by the University of California, Davis Institutional Animal Care and Use Committee, and in accordance with the guidelines proved by the National Institutes of Health for the ethical treatment of animals.

### Maternal Immune Activation.

**Figure 1:**
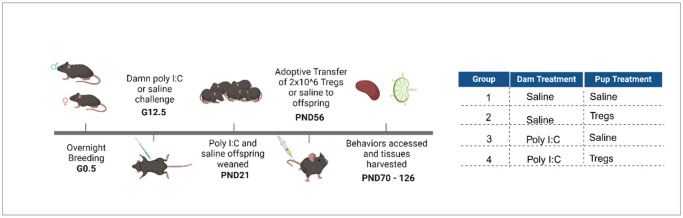
Experimental timeline and study groups. Female C57BL/6 CD45.2 mice were paired with male C57BL/6 mice, and the presence of a vaginal plug was designated as gestational day (GD) 0.5. On GD 12.5, pregnant dams received an intraperitoneal injection of either saline or 20 mg/kg Poly I:C. Dams were then allowed to deliver their offspring naturally. Regulatory T cells (Tregs) were isolated from C57BL/6 mice and expanded ex vivo for 2–3 weeks before being adoptively transferred into either MIA or saline offspring. Offspring receiving saline injections served as a control. Ten weeks after adoptive Tregs or saline administration, behavioral testing was performed, followed by collection of spleen, mesenteric lymph nodes, and brain tissues for analysis. In total, four experimental groups were evaluated (right).

9–10-week-old male and female C57 mice were paired in the evenings and females were checked every morning for the presence of seminal plugs, noted as gestational day 0.5 (G0.5) (Fig. 1). In addition, females were weighed after separation from males, and again 9–12 days later to verify pregnancy. MIA was induced in pregnant dams with polyinosinic-polycytidylic acid (poly I:C) as previously described [36]. Briefly, at gestational day 12.5, pregnant dams were injected intraperitoneally with a single dose (20 mg/kg body weight) of poly I:C (Sigma Aldrich; St. Louis, MO) or saline solution. Dams were returned to their cage and remained undisturbed until parturition. All litters appeared healthy, with no outward signs of damage due to the injections. There was no significant difference between litter sizes or between treatments. Pups remained with their mothers until postnatal day 21, when mice were weaned and housed 2–4 mice per cage with same-sex littermates.

### Regulatory T cell expansion.

8-week-old mice were anesthetized with 4% isoflurane and immediately euthanized by a pneumothorax puncture. Spleens and major lymph nodes were collected and transferred to sterile 15 mL conical tubes containing complete media: Roswell Park Memorial Institute (RPMI) 1640 media (Life Technologies) supplemented with 10% Fetal Bovine Serum (FBS), 100 IU/ml penicillin and 100 IU/ml streptomycin (Sigma) prior to processing. Spleens and lymph nodes were mechanically dissociated under aseptic conditions by using the rubber end of a sterile syringe and pressing the organ through a sterile 100 μm filter and rinsing with complete media. Cells and the media used to rinse the syringe and filter were transferred to a 50 mL conical tube, total volume was brought up to 45 mL with cold Hanks Balanced Saline Solution (HBSS) (Life Technologies) and then tubes were centrifuged for 10 mins at 500 g and 4°C. The supernatant was decanted and the cells from the lymph nodes were resuspended and washed with a second aliquot of cold HBSS and centrifuged for 10 mins at 500 g and 4°C. The supernatant was decanted again, and cells resuspended in 2 mL of ice-cold ACK lysis buffer for 1 min on ice to lyse the red blood cells. After 1 min 45 mL of ice cold HBSS was added to each tube to dilute the ACK lysis buffer, tubes were immediately placed in the centrifuge and centrifuged for 10 mins at 500 g and 4°C. After centrifugation the supernatant was decanted and cells were resuspended and washed with a second aliquot of cold HBSS, and centrifuged again for 10 mins at 500 g and 4°C. Cells were resuspended in magnetic separation buffer (AutoMACs Rinsing solution with 0.5% BSA) (Miltenyi Biotec, Auburn CA) and counted. Isolation and expansion of splenic CD4^+^CD25^+^ regulatory T cells were performed using commercially available kits from Miltenyi Biotec, following manufacturers' protocol. A negative selection was performed to separate CD4^+^ T cells from other leukocytes, followed by a positive selection to isolate CD25^+^ cells from the CD4^+^ cells. The CD4^+^CD25^+^ cells were plated at a concentration of 1 x 10^5^ cells per well in a flat bottom 96 well plate, CD3/CD28 MACSiBead particles were added at a bead to cell ratio of 3:1 in complete RPMI supplemented with 0.01 mM of 2-Mercaptoethanol and 2000 U/mL of mouse rIL-2. Expanded cells were monitored daily and cells were split/media changes were performed as suggested in the protocol. Tregs were restimulated with fresh beads every 7 days at a bead to cell ratio of 1:1. Tregs were expanded for 2–3 weeks prior to the adoptive transfer, CD3/CD28 MACSiBeads were removed using the MACSiMAG separator and washed with sterile PBS prior to the adoptive transfer. Tregs purity was confirmed by flow cytometry.

### Regulatory T cell adoptive transfer.

When offspring reached 8 weeks of age, mice were injected with 50 μL of sterile PBS or 50 μL of sterile PBS containing 2 x 10^6^ Tregs. A total of 178 offspring were injected via the tail vein (Saline: 43 males and 46 females; Tregs: 43 x males and 46 females). Saline and Tregs receiving groups were balanced within each litter so that each litter contained at least 1 male and 1 female receiving Tregs and 1 male and 1 female receiving PBS. 120 offspring [((dam treatment)saline-(pup treatment)saline females = 14, saline-saline males = 14, saline-Treg females = 14, saline-Treg males = 14, polyIC-saline females = 16, polyIC-saline males = 16, polyIC-Treg females = 16, polyIC-Treg males = 16)] were used for behavioral assays and endpoint biological assays. Any recipients of Tregs that appeared to have only partial doses (i.e., injection site leakage) were removed from analysis.

### Three-chamber social approach.

Mice were assessed for social approach behavior using the three-chamber social approach test. Each mouse was habituated for 10-minutes in the center chamber with doors closed followed by an additional 10-minutes habituation period to the entire apparatus. Habituation sessions were video recorded and analyzed to confirm a lack of innate side preference. Following habituation, experimental mice were returned to the center chamber, and a novel mouse was placed under an inverted wire cup in one side chamber and an identical empty wire cup was placed on the other side. Placement of the novel mouse or novel object (right chamber or left chamber) were randomized so that half of the mice were tested in a three-chamber apparatus that contained the novel mouse in the left chamber, and the other half were tested in a three-chamber apparatus that contained the novel mouse on the right side. Offspring were then given a 10 minutes test period and measured for the time spent in the chamber with the novel mouse and novel object. All testing chambers were thoroughly cleaned with 70% ethanol in between each testing session. Significant differences were determined using one-way ANOVAs and multiple comparisons with corrections (Holm-Sidak). Significant differences were determined if corrected p-values were < 0.05.

### Self-grooming.

To observe time spent grooming, mice were placed into a clean empty Plexiglass cage without bedding and allowed a 10-minute habituation period. Mice were video recorded for an additional 10 minutes and later scored for self-grooming behavior by individuals blind to treatment conditions. Grooming was defined as time spent licking paws, washing the nose, face or scratching fur with any foot. Significant differences were determined using one-way ANOVAs and multiple comparisons with corrections (Tukey). Significant differences were determined if corrected p-values were < 0.05.

### Elevated Plus Maze.

To determine if our experimental approach impacted anxiety like behaviors, mice underwent the elevated plus maze test. Two open arms and two closed arms extended from the central platform that was elevated approximately 1 meter from the floor. Mice were placed on the central platform and allowed to freely explore the maze for 5 minutes. The number of entries and the time spent in each closed and open arm were measured. More entries and increased time in the closed arm (and conversely fewer entries and less time in the open arms) were indicative of increased anxiety behaviors. Significant differences were determined using one-way ANOVAs and multiple comparisons with corrections (Tukey). Significant differences were determined if corrected p-values were < 0.05.

### Cellular Isolation.

After behavioral testing, offspring were euthanized and tissues were collected. Mesenteric lymph nodes (MLN) were isolated from the fat and connective tissues near the large intestine. The spleen was also collected. As above, tissues were processed by pressing through mesh and filtered, washed and then spun down in cold 1X HBSS. Cell suspensions were washed a second time and resuspended in 2 mL of complete media for counting.

### In vitro Stimulation Assays.

*In vitro* stimulations were performed on cells isolated from the MLN and spleen. 100,000 cells per well were plated in a 96 well plate Concanavalin A (10ug/mL), or phorbol myristate acetate (PMA)/Ionomycin (100 ng/mL + 1uM) were added to each well accordingly.

### Multiplex Cytokine Analysis.

Cell supernatant was collected following 48 hours *in vitro* stimulation assays and loaded onto a 96 well plate. Multiplex assay standards and quality controls were prepared following the manufacturers’ instructions. Briefly, following the protocol, the samples were incubated overnight with antibody coated magnetic beads and washed the next day. The beads were incubated with secondary antibody and then strep-PE before being run on a Bio-Plex 200 Luminex Machine. The Milliplex Mouse T_H_17 Magnetic Bead Panel (CA# MT17MAG47K-PX25) was used to access cytokine profile. The following analytes were accessed, with their assay range indicated: CD40 Ligand(CD40L) (49–50,000 pg/mL), Granulocyte-Macrophage Colony Stimulating Factor (GM-CSF)(34–35,000 pg/mL), Interferon(IFN)-γ(7.8-8,000 pg/mL), Interleukin(IL)-1β(15–15,000 pg/mL), IL-2(6.9-6,000 pg/mL), IL-4(1.5-1,500 pg/mL), IL-5(4.9-5,000 pg/mL), IL-6(7.8-8,000 pg/mL), IL-10(20–20,000 pg/mL), IL-12 (p70)(20–20,000 pg/mL), IL-13(39–40,000 pg/mL), IL-15(34–35,000 pg/mL), IL-17A(39–40,000 pg/mL), IL-17E/IL-25(586–600,000 pg/mL), IL-17F(10–10,000 pg/mL), IL-21(20–20,000 pg/mL), IL-22(2.4-2,500 pg/mL), IL-23(342–350,000 pg/mL), IL-27(879–900,000 pg/mL), IL-28B(127–130,000 pg/mL), IL-31(49–50,000 pg/mL), IL-33(78–80,000 pg/mL), Macrophage Inflammatory Protein-3 (MIP-3α/CCL20)(49–50,000 pg/mL), Tumor Necrosis Factor (TNF)-α(3.4-3,500 pg/mL), TNFβ(488–500,000 pg/mL). Cytokine concentration was determined using a standard curve of known cytokine concentrations. Each sample was run in duplicate. The observed cytokine concentration was used to test for statistically significant differences. Outliers were removed using ROUT (Q = 1%). Outlier free data was then used for downstream analysis. The non-parametric Kruskal-Wallis test with multiple comparison correction (Dunn’s) was used to determine differences between study groups.

### Flow cytometry

Using 1 x 10^6^ cells per tube, isolated cells from the MLN and spleen were washed and centrifuged twice with PBMC wash and spun at 2000 RPM for 5 minutes. Zombie Aqua Fixable Live dead stain (BioLegend) was then added at 1μl per mL and incubated for 15 minutes before washing. Cells were incubated with Fc block for 5 minutes prior to adding surface antibodies (CD3 – PercP-Cy5.5, CD45 – BV421, CD4 – AF700, CD25 – PE, Foxp3 – AF488, Rorγt – APC, Tbet – FITC) at the appropriate concentrations and incubating for 25 minutes on ice. Cells were washed and fixed with 4% paraformaldehyde before counting on a LSRII flow cytometer. Resulting data was then cleaned of outliers using ROUT (Q = 1%) and used for downstream analysis. To identify significant differences between groups, one-way ANOVAs with multiple comparison correction (Sidak) was used. Corrected p-values < 0.05 were considered statistically different.

#### RNA-Sequencing Preparation and Analysis.

From the study groups, cerebellum, cortex and hippocampus were dissected and flash frozen immediately. RNA was isolated using the Quick-DNA/RNA Miniprep Plus Kit (Zymo Research 7003T). Isolated RNA samples were treated with DNase I included the kit, to prevent DNA contamination during library preparation. RNA samples were then submitted to the UC Davis DNA Technologies Core, where RNA sample quality was determined using an Agilent Bioanalyzer System. Samples with RIN scores > 7 were used for library preparation. 3’-Tag RNA seq (Lexogen QuantSeq FWD kit, Austria) was used for library preparation. Single-end 84 Base pair reads were generated using the NextSeq 500 sequencer. Each tissue type was considered a separate library pool for batch effect and barcode purposes.

Raw sequencing reads were assessed for quality control using FastQC and multiqc, then trimmed to remove adapter contamination, polyA read through and low-quality reads using BBDuk. All samples were then re-assessed for quality using FastQC and multiqc. Following trimming and quality assessment, reads were concatenated for each sample down to a single fastq file. Samples were aligned to mouse genome (mm10) using STAR with -outFilterMultimapNmax 20 --alignSJoverhangMin 8 -- alignSJDBoverhangMin 1 --outFilterMismatchNmax 999 --outFilterMismatchNoverLmax 0.1 -- alignIntronMin 20 --alignIntronMax 1000000 --alignMatesGapMax 1000000 settings. One hippocampal sample failed initial library quality control and was removed from the analysis.

Alignment bam files were then compared using multiqc, indexed using samtools and duplicate reads removed by filtering for unique molecular indexes using umi2index. Filtered reads were counted using FeatureCounts at the gene id level with strand information using the ensmbl mm10 gene annotation. Counts were then assessed for overall quality using multiqc and read into R for statistical analysis using EdgeR and LimmaVoom. Within each brain region, to remove low-expressing genes, genes with less than 1 count per million (CPM) reads in at least 7 libraries were removed from the analysis. The remaining genes were then normalized using Trimmed Mean of M-values (TMM) to correct for library composition using calcNormFactors, method = TMM. The resulting CPM values were then fed into Voom using a design matrix for differences between pup and dam treatment conditions and sex with potential litter effects accounted for by including the dam identification number as a block in the call to Voom. Individual contrast comparisons were then called using contrasts.fit followed by eBayes. To evaluate the contribution of each factor to the overall gene expression profile, Multi-Dimensional Scaling (MDS) plots were constructed for dam treatment, pup treatment, dam and pup treatment, pup sex and dam identification (Figure S1). One frontal cortex sample was revealed as an outlier after MDS clustering and removed from further analysis. All other samples were included.

Differentially expressed genes (DEGs) were identified for each comparison of interest using the treat function without a fold change filter and the decideTests function with a false discovery rate (FDR) correction and method = separate. Significant genes were called at q < 0.05. GO term enrichments were conducted using the compareCluster function of ClusterProfiler with a q < 0.05 significance cutoff against a universe background of all genes detected in the experiment. Significant DEG lists were also examined for enrichment in previously published microglial and immune gene lists using MGEnrichment [37].

#### Weighted Gene Co-Expression Network Analysis.

Weighted Gene Co-Expression Network Analysis (WGCNA) was conducted using the WGCNA R package (version 1.68) [38, 39]. Using the reads per kilobase per million (RPKM) from the RNA-seq data, gene co-expression networks were constructed to identify modules of similarly co-expressed genes. Genes were first filtered to remove any genes with zero variance in expression or expression in fewer than 4 biological replicates. Genes were then filtered for a minimum RPKM level of 2 in at least one sample and transformed to log_2_(RPKM + 1). Genes with zero median absolute deviation were then removed, leaving 6,464 genes for analysis. Hierarchical clustering of the samples using the filtered log2RPKM values was performed and no outliers were identified (Figure S2).

Bi-weight mid-correlation between genes was used to compute a correlation matrix across all samples. Using a soft thresholding power of 5, a signed adjacency matrix, (r^2^ fit indices > 0.80) was then calculated and transformed into a topological overlap matrix (TOM). Hierarchical clustering was then performed using the matrix 1-TOM to generate co-expression modules, ensuring a minimum module size of 200 genes. Module eigengenes (ME), the first principal component of each module, were then used for statistical comparisons to identify relationships between each module and brain region, pup treatment, dam treatment, and sex using a mixed model ANOVA with a repeated measures for samples. Significant ANOVA effects or interactions for each model were followed up by Benjamini Hochberg corrected posthoc comparisons between relevant conditions.

Enrichment for gene ontology (GO) terms for each module was performed using anRichment (version 1.01-2) and anRichmentMethods (version 0.90)[38]. Genes in each module were also examined for enrichment in previously published microglial and immune gene lists using MGEnrichment [37]. A one tailed Fisher’s Exact test for enrichment of each module-gene list pair was performed with FDR correction to p < 0.05.

Hub gene analysis was performed using the WGCNA package (version 1.68). The correlations between genes within a module were calculated as intramodular connectivity. Gene module membership was calculated as the correlation between gene expression and ME. The categorical treatment conditions were then binarized to perform correlational analysis between treatment conditions and log2RPKM for gene significance. A minimum gene significance level of 0.7 and module membership value of 0.8 was used as a cut-off to identify hub genes.

## Results

### Adoptive Tregs transfer alters the frequency of peripheral and gut lymphoid T cell populations in male MIA offspring

**Figure 2:**
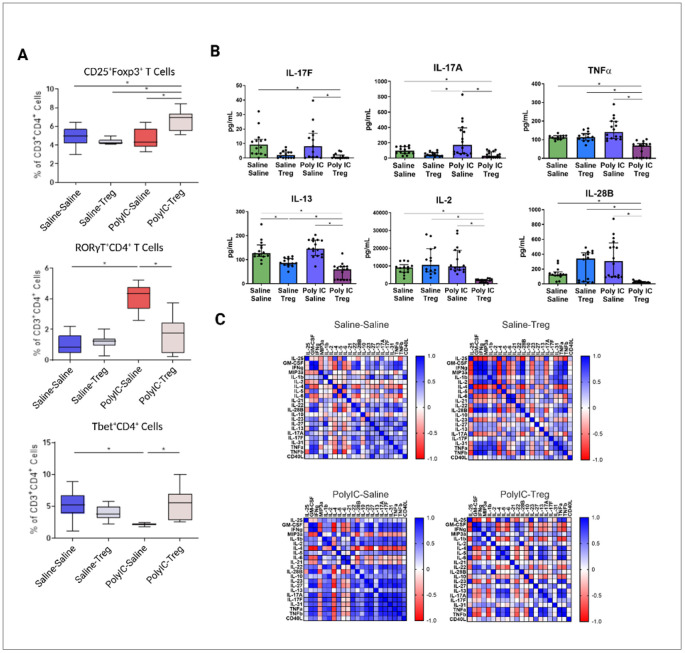
Splenic T cell populations and cytokine responses in male Poly I:C MIA offspring after adoptive regulatory T cells (Tregs) transfer. (A) In male offspring, the frequency of CD25^+^Foxp3^+^ Tregs increased following adoptive Tregs administration in the Poly I:C treated group compared to other treatment groups. Conversely, offspring exposed to Poly I:C during gestation exhibited significantly elevated frequencies of RORγT^+^ T cells but decreased after adoptive Treg treatment. (B) In male offspring, IL-17A and IL-17F were significantly decreased in the Poly I:C and Tregs group compared to Poly I:C saline and Saline-Saline controls. TNFα, IL-2, and IL-28B all show decreased levels in the Poly I:C Tregs group when compared to Saline-Saline, Saline-Tregs, and Poly I:C saline. However, there are no significant changes in these cytokines from the Saline-Saline to Poly I:C-saline groups. IL-13 is decreased in Saline-Tregs group when compared to the controls, and further decreased in Poly I:C-Saline when compared to Saline-Tregs and Poly I:C-Saline. (C) Correlations between cytokines show distinct patterns between treatment groups.

**Figure 3:**
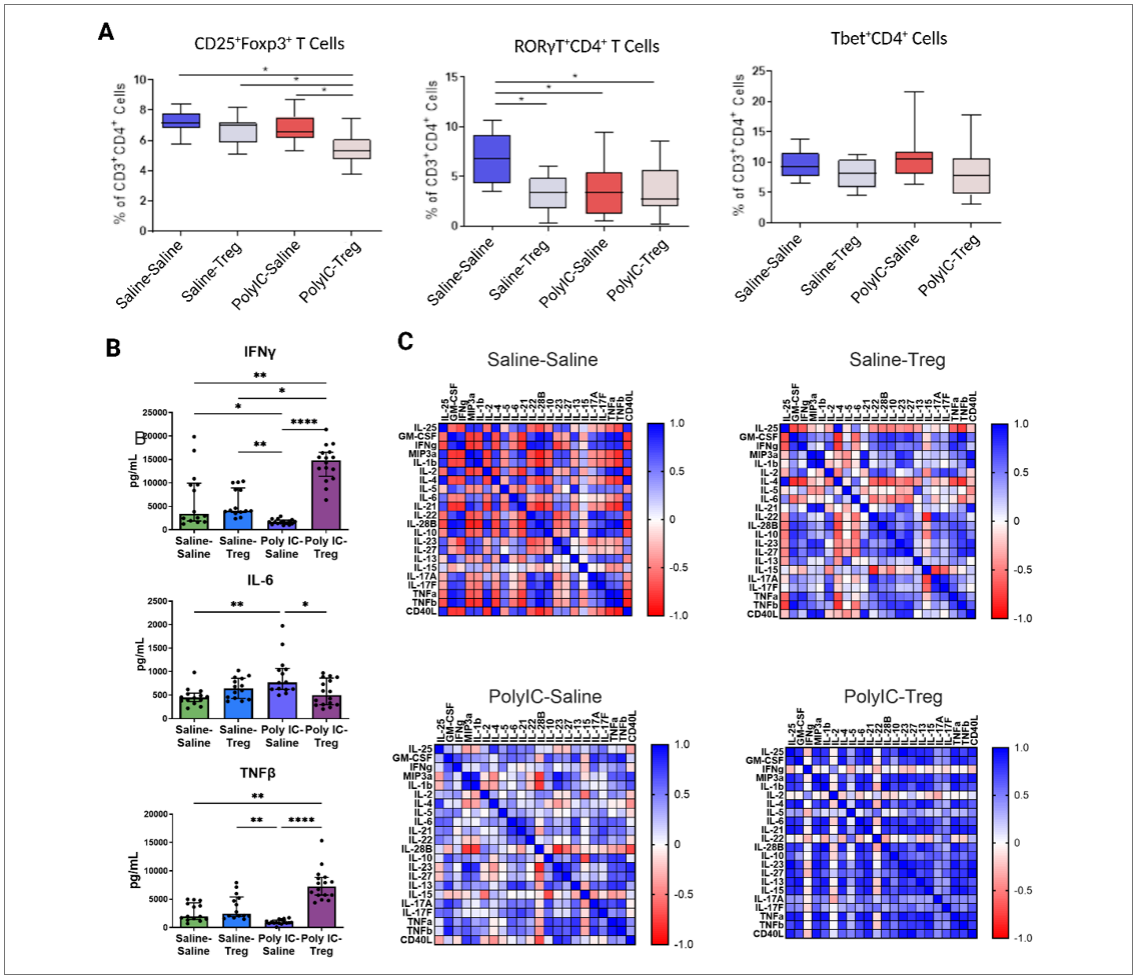
Splenic T cell populations and cytokine responses in female Poly I:C MIA offspring after adoptive regulatory T cells (Tregs) transfer. (A) In female offspring, the overall frequency of CD25^+^Foxp3^+^ Tregs was lowest in the Poly I:C group that received adoptive Tregs, compared to all other groups. Minimal differences were observed between females exposed to Poly I:C alone or adoptive Tregs alone. (B) In female offspring, a significant decrease in IFNg in the Poly IC-Saline group was seen when compared to controls. Upon addition of Tregs to the Poly I:C group, there was a significant increase in IFNg. Conversely, IL-6 was increased upon Poly I:C treatment, which decreases to control levels in the Poly I:C-Tregs group. TNFβ is significantly increased in the Poly I:C-Tregs group compared to Saline-Saline and Poly I:C-Saline. (C) Correlation analyses showed differences between Saline-Saline and Poly I:C-Tregs in correlation matrices.

To determine how adoptive transfer of Tregs influences peripheral T cell populations (CD3^+^CD4^+^), we assessed T cell phenotypes in both the spleen and MLN using flow cytometry. Firstly, we evaluated how Tregs transfer, in the context of offspring from saline treated dams (Saline-Tregs), influenced peripheral T cell populations in the spleen. No significant differences were observed in the Saline-Tregs group in male offspring compared to Saline-Saline control males in spleen (Figure 2A). However, the frequencies of RORγT^+^ (Saline-Saline: mean = 6.8%±2.5; Saline-Tregs: mean = 3.2%±1.8) and RORγT^+^Foxp3^+^ (Saline-Saline: mean = 2.8%±0.8; Saline-Tregs: mean = 1.5%±0.7) cells were decreased in the female Saline-Tregs group compared to the Saline-Saline group (Figure 3A).

We next evaluated how adoptive Tregs transfer influences peripheral populations in the context of MIA offspring. Male MIA offspring that received Tregs showed significantly increased frequencies of splenic CD25^+^Foxp3^+^ (MIA-Tregs: mean = 6.7%±1.2; MIA-Saline: mean = 4.6%±1.1) (Figure 2A) and Tbet^+^ (MIA-Tregs: mean = 7.9%±4.4; MIA-Saline: mean = 3.0%±0.4), and Tbet^+^Foxp3^+^ (MIA-Tregs: mean = 0.4%±0.4; MIA-Saline: mean = 0.1%±0.01) T cells . In contrast, RORγT^+^ T cells in male mice were significantly decreased after treatment (MIA-Tregs: mean = 3.7%±2.5; MIA-Saline: mean = 6.8%±1) (Figure 2A). In female MIA offspring only CD25^+^Foxp3^+^ T cells were different after Tregs transfer and were marginally reduced in the Tregs-treated group (mean = 4.3%±0.3) compared to untreated MIA females (mean = 4.8%±0.9) (Figure 3A).

**Figure 4:**
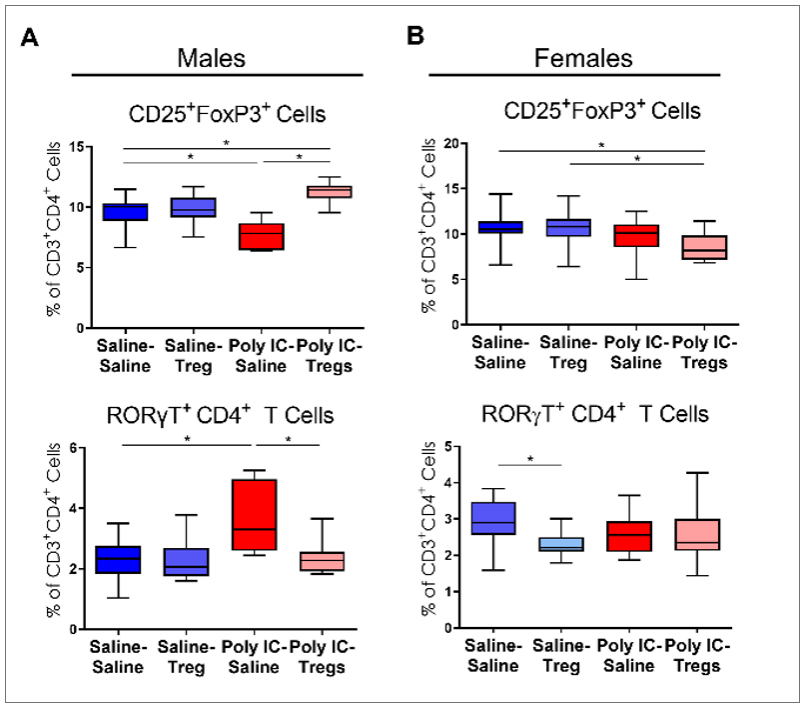
Sex specific differences in mesenteric lymph node in Poly I:C induced MIA offspring with and without adoptive regulatory T cells (Tregs) transfer. (A) In male offspring of dams exposed to Poly I:C, the frequency of CD25^+^Foxp3^+^ Tregs was decreased compared to controls but was restored following adoptive Treg transfer. RORγT^+^ T cell frequencies were elevated in Poly I:C male offspring relative to controls but were reduced in Poly I:C male offspring following adoptive Treg administration back to control levels. (B) In female offspring, mesenteric lymph node CD25^+^Foxp3^+^ Tregs frequencies were similar between Poly I:C-exposed groups and adoptive Tregs treatment alone groups. However, combined dam PolyI:C exposure and pup Tregs transfer decreased the frequency of CD25^+^Foxp3^+^ cells in females. In females, RORγT^+^T cell frequencies did not differ between the Poly I:C groups. However, compared to control females, females that received adoptive Tregs alone without prior poly I:C exposure showed significantly reduced RORγT^+^ T cell frequencies.

To examine the effect of adoptive Tregs transfer on immune populations in gut lymphoid tissue, we analyzed MLN populations via flow cytometry (Figure 4). In males, MIA offspring had lower frequencies of CD25^+^Foxp3^+^ T cells (MIA-Saline: mean = 7.7%±1.2; Saline-Saline: mean = 9.6%±1.3) and higher frequencies of RORγT^+^ T cells (MIA-Saline: mean = 7.3%±4.4; Saline-Saline: mean = 2.7%±1.1) compared to saline controls (Figure 4AB). In male MIA offspring, Tregs treatment significantly increased CD25^+^Foxp3^+^ T cells (MIA-Saline: mean = 7.7%±1.2; MIA-Tregs: mean = 11.3%±0.8) and decreased RORγT^+^ T cells (MIA-Saline: mean = 7.3%±4.4; MIA-Tregs: mean = 2.0%±0.7).

Control female offspring receiving saline (mean = 10.7%±1.7), as well as those receiving Tregs (mean 10.5%±2), had significantly more CD25^+^Foxp3^+^ T cells in the MLN than their MIA counterparts that received Tregs (mean = 8.6%±1.4) (Figure 4B). Furthermore, the effect of Tregs transfer alone showed that Tregs treatment reduced RORγT^+^ cells in female control offspring (Saline-Saline: mean = 6.7%±2.5; Saline-Tregs: mean = 3.8%±1.9) (Figure 4B). However, no reduction in RORγT^+^ T cells were observed in female MIA offspring treated with Tregs (MIA-Saline: mean = 4.1%±2.2; MIA-Tregs: 2.9%±1.7), though RORγT^+^ cells tended to be reduced after Tregs transfer.

### Adoptive Treg transfer differentially impacts cytokine production in male and female MIA offspring

Splenocytes were stimulated with PMA and ConA and supernatants analyzed via Luminex cytokine assays. In males, following PMA stimulation, a significant decrease of IL-17A and IL-17F was observed in the MIA-Tregs group when compared to the MIA-Saline group (Figure 2B). IL-13 was decreased in both Tregs groups (Saline-Tregs and MIA-Tregs) when compared to their counterparts after PMA stimulation. In females, IL-6 was significantly increased in MIA offspring compared to control offspring and was decreased upon adoptive transfer of Tregs (Figure 3B). PMA stimulated IFNy was also decreased in female MIA offspring compared to controls and significantly increased upon Tregs treatment.

Correlation analysis between all readable cytokines in the male offspring, revealed several patterns between treatment groups (Figure 2C). The MIA-Saline group showed positive correlations between cytokines like IL-17A, IL-17F, IL-31, TNFα, and TNFβ. These correlations were weak in Saline-Saline controls. In MIA-Tregs group cytokine correlations closely resemble the patterns observed in the Saline-Saline controls, suggesting a shift towards a more homeostatic baseline upon addition of the Tregs. However, strong positive correlations between TNFα, TNFβ, and IL-17A persisted in the MIA-Tregs group. Interestingly, the Saline-Tregs group showed a differing profile from the Saline-Saline controls, with several strong positive correlations seen between IFNγ, MIP-3a, and GM-CSF where previously these had been weak negative correlation with each other. In females, Saline-Saline offspring had many cytokines that were strongly negative correlated to each other, whereas the MIA-Tregs group showed mainly strong positive correlations (Figure 3C). Saline-Tregs and MIA-Tregs showed similar patterns of weak correlations between many of the cytokines.

After ConA stimulation, an increase in IL-6 was seen in the male MIA offspring, and was rescued by Tregs transfer (Supplemental S1). The female splenocytes showed increased IL-2 and IL-6 in both MIA-Saline and MIA-Treg compared to controls, with no impact of Tregs on cytokine changes in the MIA group in female offspring (Figure S2).

### Adoptive regulatory T cell transfer induced differential gene expression in the brain of male control and MIA offspring

We identified DEGs within each brain region using a full statistical model for the interaction of dam treatment, pup treatment and sex while controlling for litter effects (Table S1). In the cerebellum, 246 genes showed increased expression and 388 showed decreased expression in offspring treated with Tregs from Saline compared to MIA treated dams.

**Figure 5:**
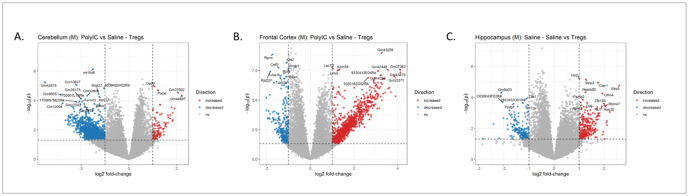
Gene expression between brain regions differs between male Poly I:C offspring who were treated with saline or adoptive Tregs. 3’-Tag RNA sequencing was performed to identify DEGs in the (A) cerebellum, (B) frontal cortex, and (C) hippocampus. A total of 280 DEGs were detected in the cerebellum, 2,586 in the frontal cortex, and 421 in the hippocampus.

When separated by sex, no DEGs were identified in females. In males, we identified 119 DEGs with lower levels of expression in male offspring from MIA-Tregs dams compared to MIA- saline (Figure 5A). Similarly, there were 161 genes with higher expression between these same conditions. Genes with higher expression in male offspring from MIA-Tregs compared to saline treated dams were enriched for gene ontology (GO) terms related to transcription regulation and synaptic transmission (Table S1). Using MGEnrichment application that contains a database of mouse and human microglial studies [37], we identified significant enrichments between these same genes and genes related to several neurodevelopmental disorders including ASD (Table S1). These sets of male DEGs and non-sex specific DEGs were mostly, but not completely, overlapping (Figure S5), suggesting similar but more subtle changes in gene expression upon Tregs treatment in females compared to males.

In the frontal cortex there were 1,128 genes with increased expression and 1,458 genes with decreased expression between Tregs treated male offspring from saline and MIA treated dams (Figure 5B). This difference was not observed in the female offspring nor in any other treatment comparison in the males. Genes with increased expression were enriched for GO terms involving histone regulation, chromatin regulation, synapse formation and actin cytoskeleton regulation. They were also enriched for MGEnrichment lists for ASD related genes and genes differentially expressed in microglia from offspring whose dams were exposed to maternal allergic asthma in utero (MAA microglial DEGs). Genes with decreased expression were enriched for GO terms for mitochondrial function and translation regulation as well as MGEnrichment for microglial oxidative phosphorylation, proliferation and phagocytosis. Collapsing across sex revealed 281 DEGs with increased expression and 337 with decreased expression between male offspring from saline and MIA treated dams that were given Tregs. These DEGs were almost completely composed of a subset of the DEGs identified uniquely in the males for the same comparison (Figure S6). This decrease in the number of significant DEGs when including males and females together in this comparison highlights the differences in response between the two sexes. Together, this suggests that treatment with Tregs had differential impacts on frontal cortex gene expression depending on the *in utero* experience and that this response was driven by changes in males.

In comparison, in the female offspring but not males, there was a single gene increased in expression and two with decreased expression between offspring given saline and Tregs from dams treated with MIA. This difference was not observed in the males nor in any other treatment comparison in the females. Collapsing across sex increased the number of DEGs identified between offspring treated with saline versus Tregs from MIA treated dams (10 increased and 16 decreased in expression respectively), suggesting similar but more subtle changes in males under these conditions.

In the hippocampus, the only significant differences were in male offspring. There were 300 DEGs with higher expression and 121 with lower expression in offspring treated with saline compared to Tregs, from dams treated with saline (Figure 5C). These differences did not appear in any other condition nor in females. Genes with increased expression were enriched for MGEnrichment gene lists related to neurological disorders, ASD, and microglial immune responses. Genes with decreased expression were enriched for GO terms related to neuronal synapses, neurotransmission and several neurological diseases. They were also enriched for MGEnrichment lists involving several brain disorders including ASD, MAA microglial DEGs, and several microglial states related to immune regulation. Collapsing across sex, did not reveal any significant DEGs, suggesting the impacts of dam and offspring treatments on hippocampal gene expression was unique to male offspring.

### Weighted Gene Co-Expression Network Analysis Reveals Sex- and Region-Specific Gene Networks Altered by Early-Life Inflammation and Tregs Treatment

**Figure 6:**
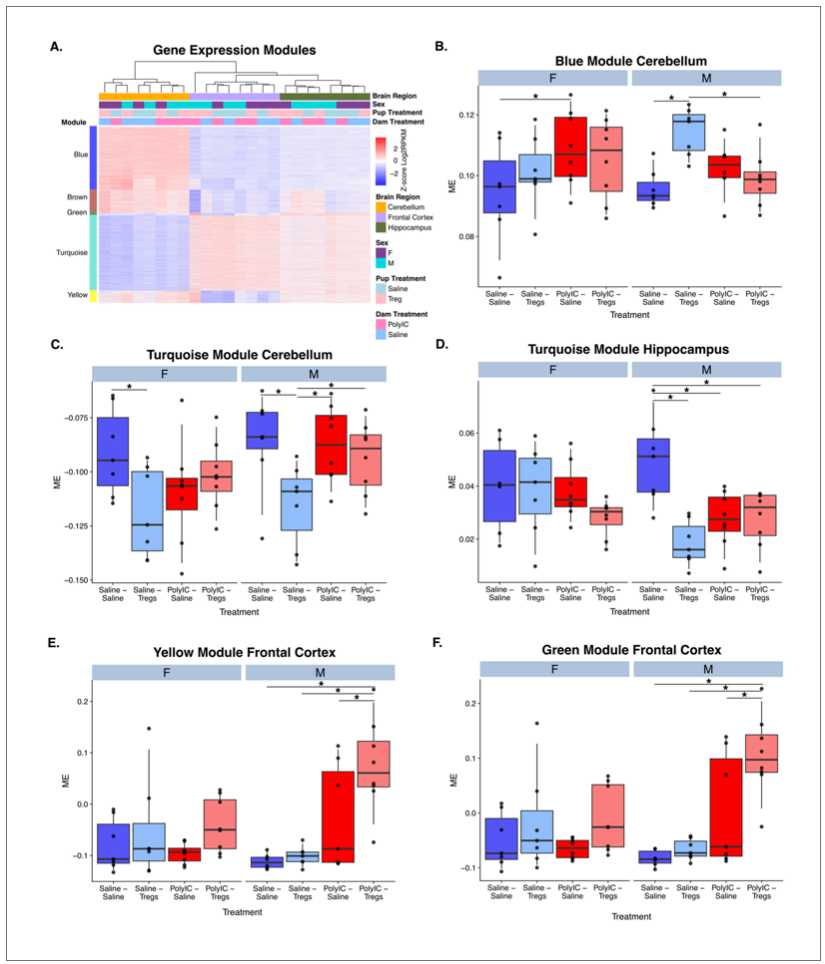
Weighted gene co-expression network analysis (WGCNA) identifies modules of genes altered by early-life inflammation and Tregs treatment in a sex- and region-specific manner. (A) Expression of genes in each of the five co-expression modules (blue, turquoise, brown, green, yellow). Log2RPKM values are Z scored by row. (B–F) Module eigengene (ME) values for each module analyzed by repeated-measures ANOVA with dam treatment, pup treatment, sex, and brain region as factors. Post hoc Benjamini–Hochberg corrected comparisons highlight significant effects of Tregs and PolyI:C treatments across brain regions. (B) Blue module: Tregs increased ME in male saline-treated pups, an effect blocked by maternal Poly I:C exposure. (C-D) Turquoise module: significant pup and dam treatment effects in cerebellum and hippocampus, predominantly in males. (E–F) yellow and green modules in the frontal cortex: males show increased in Poly I:C-Tregs groups compared to all others. All modules were significant for brain region (p < 0.05; Table S2). Error bars represent ± SEM.

To further explore patterns of gene expression modulated by early life inflammation and later life Tregs treatment, we conducted a weighted gene co-expression network analysis (WGCNA) and identified 5 modules (blue, turquois, brown, green, yellow) of co-expressed genes(Figure S4). Each of these modules serve as a cluster of interconnected genes with similar expression patterns, suggesting a shared functionality. We conducted a repeated measures ANOVA for each set of module eigengenes (ME) using dam treatment, pup treatment, sex and brain region as factors (Figure 6 and S3). To further explore these interactions, we conducted a series of Benjamini Hochberg corrected posthoc comparisons on the module eigengenes between offspring and dam treatment conditions within each brain region and sex (Figure 6A-F). All five modules were significant for brain region (p < 0.05, Table S2).

The blue module was significant for the main effect of offspring treatment and interactions between offspring treatment and brain region; offspring treatment and dam treatment; and offspring treatment, dam treatment and brain region. These effects were largely driven by changes within the cerebellum. Posthoc comparisons revealed a significant increase in the blue ME for male Saline-Tregs pups compared to Saline-Saline pups. The male Saline-Tregs pups were also significantly higher than male MIA-Treg pups, and male MIA-Saline was greater than male Saline-Saline (Figure 6B and Table S2). This indicates that genes in the blue module were significantly impacted by Tregs and that this effect was blocked if the dam received MIA induced by PolyI:C. This effect was also unique to males. Genes in the blue module were significantly enriched for four different gene lists related to Chd8 regulation including target genes of Chd8 binding and genes that are differentially expressed in brains of Chd8 heterozygous knockout mice (Table S2). The blue module was also enriched for genes involved in microglial metabolism, cellular stress and proliferation (Table S2). Together this indicates that Tregs impact expression of genes related to Chd8 regulation and microglial function in the brain and that these processes are disrupted in MIA male offspring.

The turquoise module was significant for the main effect of offspring treatment and the interactions between offspring treatment and brain region; dam treatment and offspring treatment; and the three-way interaction between dam treatment, brain region and sex (Table S2). Posthoc tests revealed significant impact in both the cerebellum and hippocampus. In the cerebellum, both males and females showed a significant decrease in module ME between Saline-Saline and Saline-Tregs treated offspring. Males also showed significant differences between Saline-Saline and MIA-Saline, Saline-Tregs and MIA-Tregs, and Saline-Tregs and MIA-Saline. In the hippocampus, the turquois ME showed a similar pattern with significant differences between Saline-Saline and Saline-Tregs that was lost in the MIA-Saline compared to MIA-Tregs (Figure 6C). Together these differences in males reflect a similar pattern as those observed in the blue module where Tregs treatment has an impact in saline treated dams that is lost in MIA treated dams (Figure 6D). These patterns are similar but less well delineated in female offspring. The turquois model is enriched for GO terms related to neuronal function, synapses, dendrites, and synaptic transmission. The turquoise module was also enriched for Chd8 target genes and numerous lists related to microglial regulation including proliferation, microbiome responses, inflammatory responses and development (Table S2).

The brown, green and yellow modules were all significant for the interaction between brain region and dam treatment. Further posthoc analysis of both the yellow (Figure 6E) and green (Figure 6F) modules, found a significant increase in ME for MIA-Tregs compared to all other groups in the frontal cortex. The only significant posthoc difference in the brown module was a decrease in ME in the MIA-Tregs compared to Saline-Tregs conditions. The yellow module was enriched in GO terms related to transcription, including RNA processing, RNA splicing, and mRNA metabolic processing. The green module did not have any significant GO terms associated with it. The brown module consisted of GO terms related to mitochondrial and ribosomal processes, including “cytosolic ribosome”, “mitochondrial protein complex”, and “oxidation-reduction process” (Figure S4 and Table S2).

### Adoptive Tregs transfer influences social approach and self-grooming in MIA offspring in sex dependent manner

**Figure 7:**
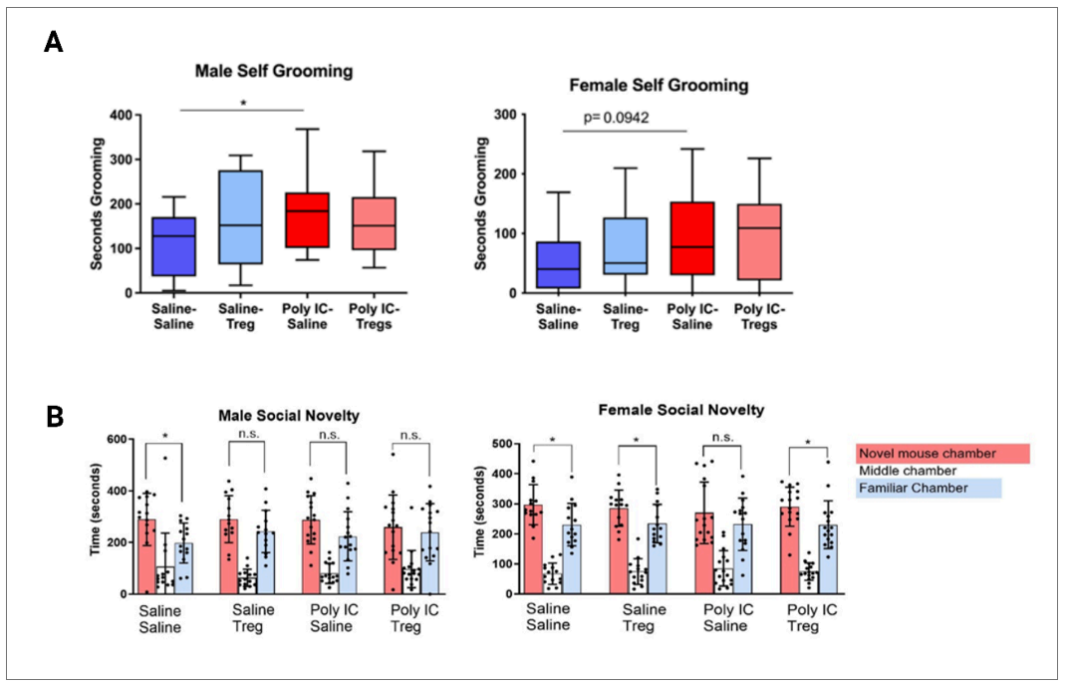
Offspring of MIA treated dams exhibit sex specific differences in repetitive and social behaviors. (A) Male offspring exposed to Poly I:C during gestation (n = 15) exhibited increased self-grooming behavior compared to saline-exposed males (n = 13). A similar trend was observed in female offspring exposed to Poly I:C (n = 16) compared to saline controls (n = 14), although the difference did not reach statistical significance. After Tregs adoptive transfer in offspring of Poly I:C exposed dams there were no differences over saline controls. (B) Female offspring exposed to Poly I:C showed no preference between chambers containing a novel versus familiar mouse, while Poly I:C-exposed females that received Tregs demonstrated a significant preference for the novel mouse chamber. Group comparisons were conducted using ordinary one-way ANOVAs and unpaired t-tests. Statistical significance was defined as *p* < 0.05.

To assess behaviors in the MIA model previously described by us and others [6, 40], grooming, social and anxiety behaviors were measured. Male MIA-Saline offspring exhibited significantly more self-grooming behaviors (176.7 seconds±79.9) than the Saline-Saline control counterparts (107.8 seconds±70.7, p = 0.028) (Figure 7A). However, male offspring from MIA-Tregs group did not show any difference in self-grooming behavior compared to Saline-Saline controls (158.4 seconds±75.1) (p = 0.11). In female MIA-Saline offspring, there was also a tendency to have increased self-grooming behaviors (96 seconds±72.9) compared to their respective controls (70.1 seconds±74.2) but this did not reach statistical significance (p = 0.094) (Figure 7A).

To assess social novelty, a 3-chamber social test was administered. Female Saline-Saline mice spent more time (mean = 297.2s, SD = 66.4) with the novel mouse than the familiar chamber (mean = 230.9s, SD = 70.6). In female MIA-Saline, social novelty was not significantly different in their interaction times between novel (mean = 270s, SD = 102s) and familiar mouse (mean = 232s, SD = 87s) chambers suggesting decreased social novelty behaviors (Figure 7B). However, after Tregs treatment, female MIA offspring exhibited similar social novelty compared to the Saline-Saline control group, with a preference for the novel mouse (mean = 290.4s, SD = 65.2s) compared to the familiar chamber (mean = 230.3s, SD = 79.4s). In male Saline-Saline offspring, significantly more time was spent within the novel mouse chamber (mean = 289.4s, SD = 101.1s) compared to the familiar mouse chamber (mean = 197.9s, SD = 77.5s). This was not observed in male MIA-Saline mice, which trended towards having a preference towards the familiar mouse chamber (mean = 223.9, SD = 95.4) over the novel mouse chamber (mean = 287.8s, SD = 92.9s) but did not reach significance. Male MIA-Tregs offspring trended towards a reduced amount of time spent in the novel mouse chamber (mean = 259.2s, SD = 124.5s) over the familiar mouse chamber (mean = 239.5, SD = 110.7s), but did not reach statistical significance either.

The impact of MIA on long term memory was evident between male control Saline-Saline offspring and MIA-Saline offspring, without Tregs transfer. In male control offspring receiving saline, the median time spent with the moved object was greater (mean = 10.73s, SD = 6.95) than male MIA-Saline offspring (mean = 4.99s, SD = 4.4, p = 0.052). No differences were observed across other study groups, though OLM tended to be lower in both MIA offspring groups (MIA- Tregs = 4.99s, SE = 4.4; MIA -Saline = 5.6s, SE = 4.1) in comparison to the saline groups, Saline-Tregs (mean = 10.7, SE = 6.9) or Saline-Saline (mean = 7.04s, SE = 5.5). No significant differences were observed in female study groups.

## Discussion

The therapeutic application of Tregs to ameliorate NDD related behaviors is an innovative approach rooted in the growing recognition of chronic inflammation as a key feature in these conditions. In this study, we assessed how adoptive Tregs transfer influences the immune response, brain gene expression and behavioral phenotypes of offspring born to dams exposed to an inflammatory challenge during gestation. Adoptive transfer of Tregs resulted in several immunological changes in MIA offspring in a sex-dependent manner. In male MIA-Saline offspring, we observed increased peripheral and gut T_H_17 cells, as well as reduced frequencies of Tregs. After Tregs transfer, the frequency of T_H_17 cells in spleen and lymphoid tissues was reduced. This was not observed in the female MIA-Saline offspring, evidenced by no clear increase in peripheral and gut T_H_17 populations. To determine whether Tregs transfer also impacted neurobiological processes, we assessed RNA transcriptomic profiles in brain regions commonly implicated in NDDs behaviors. Consistent with our immunological findings, the most notable transcriptomic changes occurred in male MIA offspring across the cerebellum, frontal cortex, and hippocampus. Differentially expressed genes were enriched in pathways related to transcriptional regulation, epigenetic processes, and synaptic neurotransmission, respectively. Lastly, we observed sex specific changes in behavior after Tregs transfer. In summary, adoptive Tregs transfer exerted sex-specific effects on immune function, transcriptomics in the brain, and behavioral outcome in the context of MIA.

Clinical and preclinical studies of NDDs suggest that immune dysregulation may contribute to their etiology, with immune imbalance typically associated with worse behavioral outcomes [41-43]. In NDDs such as ASD, increased peripheral inflammatory cytokines and immune cells are commonly reported, including those involved in T cell biology [14]. For example, elevated frequencies of T_H_17 cells, defined by their expression of the master transcription factor RORγT and production of the pro-inflammatory cytokine IL-17A, are increased the peripheral blood of children with ASD and in preclinical models [12, 23, 24, 44, 45]. In the current study, we also find increased T_H_17 cells in both peripheral and gut lymphoid tissue in MIA males. Tregs play a pivotal role in maintaining immune system homeostasis and controlling inflammation, and also control T_H_17 mediated inflammation. Tregs and their canonical cytokines are often reduced in ASD [24, 45-48]. In this study we also found lower Tregs in offspring after MIA. This imbalance between T_H_17 cells and Tregs may be explained by their developmental plasticity, as inflammatory environments can drive the peripheral conversion of Tregs towards T_H_17 phenotypes [49].

In male MIA offspring, adoptive transfer of donor Tregs reduced RORγT^+^ T cell frequencies and restored them to the frequencies observed in saline controls, in both spleen and intestinal lymphoid tissues. In addition, Tregs transfer increased the frequency of peripheral host Tregs and Tbet^+^ CD4^+^ T cells in these males. Tbet is the master transcription factor for T_H_1 cells, which are classically involved in immunity against intracellular pathogens. T_H_1 cells and their signature cytokine, IFNγ, have also been implicated in promoting social behaviors [50]. Moreover, Tbet^+^ Tregs have been shown to mediate protective effects in multiple mouse models, including crescentic glomerulonephritis and other type 1 inflammation-driven conditions [51, 52]. These effects, however, were only observed in males. In females, Saline-Saline animals already exhibited higher baseline frequencies of T_H_17 cells, and adoptive Tregs transfer led to only a modest reduction in T_H_17 frequencies and did not alter Tbet^+^ populations. It is possible that the changes in T_H_17 cells in male MIA-Saline offspring are more observable in pathogenic T_H_17 populations, rather than homeostatic T_H_17 populations, due to increased IL-23 production after PMA splenocyte stimulation, a key factor in pathogenic T_H_17 cell development [53, 54]. This raises the possibility that underlying immune pathology differs between male and female in MIA offspring and may explain why adoptive Tregs transfer was more effective in one sex but not the other. In female MIA offspring, Tregs transfer led to a reduction in Tregs frequencies compared to all other groups. Interestingly, cytokine profiles also differed based on sex between Tregs-treated and untreated offspring. Generally, in female MIA-Tregs offspring, cytokine secretion of pro-inflammatory cytokines was increased after PMA stimulation, exacerbating the already higher cytokine levels observed in female MIA-Saline offspring. The opposite was true for males, where male MIA-Saline had increased cytokine production upon PMA stimulation but was generally lower in male MIA-Tregs offspring. These differences in cytokine and cellular profiles between male and female MIA offspring under Tregs treatment conditions may explain why behaviors, particularly self-grooming, and long-term memory, were more pronounced in male MIA offspring than female MIA offspring. Using Tregs as a therapeutic avenue therefore may have reshaped immune networks in a sex-specific manner.

The brain is sensitive to changes in immune status. Inflammation can directly alter neurotransmission, disrupting the balance between inhibitory and excitatory neuronal signaling and impacting the activation status of resident immune cells, particularly microglia [50, 55-59]. In ASD, studies have identified altered neural connectivity, pro-inflammatory microglial activation, and changes in the synaptic compartment [60, 61]. However, much less is known about how treatments targeting the immune system affect the central nervous system (CNS). Changes in peripheral and lymphoid immune cell frequencies may influence the transcriptional profile of various brain regions. This is evident in our finding of a high number of DEGs in male MIA offspring compared to untreated males, whereas female MIA offspring showed little to no DEG changes, regardless of treatment also suggesting sex-differences in MIA responses.

Among the DEGs enriched in the brains of male MIA offspring, regardless of brain region or direction of expression, most related to synaptic function or gene expression regulation at the transcriptional or translational level. Although all three brain regions examined (frontal cortex, cerebellum, and hippocampus) showed changes, the frontal cortex appeared the most responsive to both MIA and subsequent Tregs treatment, exhibiting nearly tenfold more DEGs than the cerebellum or hippocampus. The frontal cortex is critical for higher-order processes including cognition, emotional regulation, and executive decision-making [62]. Its dysfunction is well established in ASD, as common ASD-associated behaviors such as social impairment and deficits in executive function are strongly linked to frontal lobe activity. Supporting this, frontal lobe volume is often increased in individuals with ASD, and a recent imaging study found that adult males with ASD displayed more impaired frontal lobe white matter connectivity than their female counterparts [63, 64]. One possible mechanism by which Tregs influenced the frontal cortex is through activation of members of the TGF family, which are produced by resident glial cells [65]. TGF family members (TGFβ1, TGFβ2, and TGFβ3) are typically secreted in a latent, inactive form and, when activated, and are known to be involved in blood vessel morphogenesis and synaptic development [66, 67]. Tregs are a significant producer of TGFβ in the immune system, therefore, it is possible that adoptive Tregs transfer altered gene expression in the brain via activation of TGF signaling pathways. Moreover, the effects may be specific to males, as this study observed few transcriptomic changes in female offspring, regardless of Tregs treatment.

While adoptive transfer of Tregs is a promising therapeutic approach, this study has several limitations that future research should consider. First, the experimental time points for adoptive transfer of Tregs occurred in adult offspring at 10 weeks of age. It is possible that a different therapeutic effect, particularly on behavior, could be achieved by transferring Tregs into younger mice, during a period when the immune system and brain are still co-developing. Therefore, the timing used in this current study may be more relevant for addressing MIA-related comorbidities, such as inflammation, rather than for modifying core behavioral symptoms. Second, we identified minimal immunological or neurological effects of Tregs transfer in female MIA offspring, whereas in male MIA offspring, we generally found improved immune status after treatment. Therefore, our study highlights an important sex difference regarding potential mechanism lacking in other studies utilizing Tregs or Tregs targeted approaches. It is possible that Tregs are not necessary for treating the specific pathological mechanisms driving the MIA phenotype in females, or that a different immunological pathway is more relevant in female offspring. Lastly, while this study primarily focused on how adoptively transferred Tregs influence T cell biology in the context of ASD, we did not investigate underlying immune mechanisms in depth such as those involving the innate immune system, nor did we assess additional T cell subsets beyond Tregs and T_H_17. Future studies should examine other immune populations to build a more complete picture of how Tregs regulate immune dysfunction in MIA.

In conclusion, the present study supports a beneficial role for adoptive Tregs transfer in the MIA model, particularly for immune-related dysfunction in male offspring. These findings may offer important insights into how Tregs-based therapies could translate into clinical practice for treating systemic inflammation and select comorbidities in ASD.

## Supplementary Material

This is a list of supplementary files associated with this preprint. Click to download.

- SFigure1.docx
- SFigure2.docx
- TableS2.xlsx
- SFigure36.docx
- TableS1.xlsx
- SupplementalTable2legend.docx
- SupplementalTable1legend.docx

## References

[1] Maenner M.J., , Prevalence and Characteristics of Autism Spectrum Disorder Among Children Aged 8 Years — Autism and Developmental Disabilities Monitoring Network, 11 Sites, United States, 2020. MMWR Surveillance Summaries, 2023. 72(2): p. 1–14. 10.15585/mmwr.ss7202a1 PMC10042614 36952288

[2] Tamayo J.M., , The influence of asthma on neuroinflammation and neurodevelopment: From epidemiology to basic models. Brain, Behavior, and Immunity, 2024. 116: p. 218–228. 38070621 10.1016/j.bbi.2023.12.003

[3] Careaga M., Murai T., and Bauman M.D., Maternal Immune Activation and Autism Spectrum Disorder: From Rodents to Nonhuman and Human Primates. Biological Psychiatry, 2017. 81(5): p. 391–401. 28137374 10.1016/j.biopsych.2016.10.020 PMC5513502

[4] Croen L.A., , Maternal Autoimmune Diseases, Asthma and Allergies, and Childhood Autism Spectrum Disorders: A Case-control Study. Archives of Pediatrics & Adolescent Medicine, 2005. 159(2): p. 151–157. 15699309 10.1001/archpedi.159.2.151

[5] Osman H.C., , Impact of maternal immune activation and sex on placental and fetal brain cytokine and gene expression profiles in a preclinical model of neurodevelopmental disorders. Journal of Neuroinflammation, 2024. 21(1): p. 118. 38715090 10.1186/s12974-024-03106-7 PMC11077729

[6] Malkova N.V., , Maternal immune activation yields offspring displaying mouse versions of the three core symptoms of autism. Brain, Behavior, and Immunity, 2012. 26(4): p. 607–616. 22310922 10.1016/j.bbi.2012.01.011 PMC3322300

[7] Smith S.E.P., , Maternal Immune Activation Alters Fetal Brain Development through Interleukin-6. Journal of Neuroscience, 2007. 27(40): p. 10695–10702. 17913903 10.1523/JNEUROSCI.2178-07.2007 PMC2387067

[8] Choi G.B., , The maternal interleukin-17a pathway in mice promotes autism-like phenotypes in offspring. Science, 2016. 351(6276): p. 933–939. 26822608 10.1126/science.aad0314 PMC4782964

[9] Rose D.R., , Long-term altered immune responses following fetal priming in a non-human primate model of maternal immune activation. Brain, Behavior, and Immunity, 2017. 63: p. 60–70. 27876552 10.1016/j.bbi.2016.11.020 PMC5432383

[10] Onore C., , Dynamic Akt/mTOR Signaling in Children with Autism Spectrum Disorder. Frontiers in Pediatrics, 2017. 5: p. 43. 28361047 10.3389/fped.2017.00043 PMC5350147

[11] Boktor J.C., , Global metabolic profiles in a non-human primate model of maternal immune activation: implications for neurodevelopmental disorders. Molecular Psychiatry, 2022. 27(12): p. 4959–4973. 36028571 10.1038/s41380-022-01752-y PMC9772216

[12] Mandal M., , Preferential development of Th17 cells in offspring of immunostimulated pregnant mice. Journal of Reproductive Immunology, 2010. 87(1-2): p. 97–100. 20727596 10.1016/j.jri.2010.06.156

[13] Hughes H.K., Moreno R.J., and Ashwood P., Innate immune dysfunction and neuroinflammation in autism spectrum disorder (ASD). Brain, Behavior, and Immunity, 2023. 108: p. 245–254. 36494048 10.1016/j.bbi.2022.12.001

[14] Moreno R.J., Amara R.A., and Ashwood P., Toward a better understanding of T cell dysregulation in autism: An integrative review. Brain, Behavior, and Immunity, 2025. 123: p. 1147–1158. 39378971 10.1016/j.bbi.2024.10.009

[15] Runge K., , Altered cytokine levels in the cerebrospinal fluid of adult patients with autism spectrum disorder. Journal of Psychiatric Research, 2023. 158: p. 134–142. 36584491 10.1016/j.jpsychires.2022.12.032

[16] Vargas D.L., , Neuroglial activation and neuroinflammation in the brain of patients with autism. Annals of Neurology, 2005. 57(1): p. 67–81. 15546155 10.1002/ana.20315

[17] Tomaiuolo P., , RNA sequencing of blood from sex- and age-matched discordant siblings supports immune and transcriptional dysregulation in autism spectrum disorder. Scientific Reports, 2023. 13(1): p. 807. 36646776 10.1038/s41598-023-27378-w PMC9842630

[18] Ashwood P. and Wakefield A.J., Immune activation of peripheral blood and mucosal CD3+ lymphocyte cytokine profiles in children with autism and gastrointestinal symptoms. Journal of Neuroimmunology, 2006. 173(1-2): p. 126–134. 16494951 10.1016/j.jneuroim.2005.12.007

[19] Dikiy S. and Rudensky A.Y., Principles of regulatory T cell function. Immunity, 2023. 56(2): p. 240–255. 36792571 10.1016/j.immuni.2023.01.004

[20] Rose D. and Ashwood P., Rapid Communication: Plasma Interleukin-35 in Children with Autism. Brain Sciences, 2019. 9(7): p. 152. 31252635 10.3390/brainsci9070152 PMC6680732

[21] Jyonouchi H., Geng L., and Davidow A.L., Cytokine profiles by peripheral blood monocytes are associated with changes in behavioral symptoms following immune insults in a subset of ASD subjects: an inflammatory subtype? Journal of Neuroinflammation, 2014. 11(1): p. 187. 25344730 10.1186/s12974-014-0187-2 PMC4213467

[22] Ashwood P., , Decreased transforming growth factor beta1 in autism: A potential link between immune dysregulation and impairment in clinical behavioral outcomes. Journal of Neuroimmunology, 2008. 204(1-2): p. 149–153. 18762342 10.1016/j.jneuroim.2008.07.006 PMC2615583

[23] Ahmad S.F., , Dysregulation of Th1, Th2, Th17, and T regulatory cell-related transcription factor signaling in children with autism. Molecular Neurobiology, 2017. 54(6): p. 4390–4400. 27344332 10.1007/s12035-016-9977-0

[24] Rose D.R., , T cell populations in children with autism spectrum disorder and co-morbid gastrointestinal symptoms. Brain, Behavior, & Immunity - Health, 2020. 2: p. 100042. 10.1016/j.bbih.2020.100042 PMC8474588 34589832

[25] Schweingruber N., , Mechanisms of Glucocorticoids in the Control of Neuroinflammation. Journal of Neuroendocrinology, 2012. 24(1): p. 174–182. 21615563 10.1111/j.1365-2826.2011.02161.x

[26] Ros-Bernal F., , Microglial glucocorticoid receptors play a pivotal role in regulating dopaminergic neurodegeneration in parkinsonism. Proceedings of the National Academy of Sciences, 2011. 108(16): p. 6632–6637. 10.1073/pnas.1017820108 PMC3080980 21467220

[27] Shenoy S., Arnold S., and Chatila T., Response to steroid therapy in autism secondary to autoimmune lymphoproliferative syndrome. The Journal of Pediatrics, 2000. 136(5): p. 682–687. 10802504 10.1067/mpd.2000.105355

[28] Mordekar S.R., , Corticosteroid treatment of behaviour, language and motor regression in childhood disintegrative disorder. European Journal of Paediatric Neurology, 2009. 13(4): p. 367–369. 18625572 10.1016/j.ejpn.2008.06.001

[29] Stefanatos G.A., Grover W., and Geller E., Case Study. Journal of the American Academy of Child & Adolescent Psychiatry, 1995. 34(8): p. 1107–1111. 7545148 10.1097/00004583-199508000-00022

[30] Xu Z., , Rescue of maternal immune activation-induced behavioral abnormalities in adult mouse offspring by pathogen-activated maternal Treg cells. Nature Neuroscience, 2021. 24(6): p. 818–830. 33859437 10.1038/s41593-021-00837-1

[31] Li M., , LdIL-2 treatment in ASD: a novel immunotherapeutic approach targeting Th/Treg dysfunction and neuroinflammation. Translational Psychiatry, 2025. 15(1): p. 376. 41053009 10.1038/s41398-025-03609-8 PMC12500982

[32] Ahmad S.F., , S3I-201, a selective Stat3 inhibitor, restores neuroimmune function through upregulation of Treg signaling in autistic BTBR T+ Itpr3tf/J mice. Cellular Signalling, 2018. 52: p. 127–136. 30213685 10.1016/j.cellsig.2018.09.006

[33] Lv Y.-T., , Transplantation of human cord blood mononuclear cells and umbilical cord-derived mesenchymal stem cells in autism. Journal of Translational Medicine, 2013. 11(1): p. 196. 23978163 10.1186/1479-5876-11-196 PMC3765833

[34] Dawson G., , Autologous Cord Blood Infusions Are Safe and Feasible in Young Children with Autism Spectrum Disorder: Results of a Single-Center Phase I Open-Label Trial. Stem Cells Translational Medicine, 2017. 6(5): p. 1332–1339. 28378499 10.1002/sctm.16-0474 PMC5442708

[35] Dawson G., , A Phase II Randomized Clinical Trial of the Safety and Efficacy of Intravenous Umbilical Cord Blood Infusion for Treatment of Children with Autism Spectrum Disorder. The Journal of Pediatrics, 2020. 222: p. 164–173.e5. 32444220 10.1016/j.jpeds.2020.03.011

[36] Giulivi C., , Gestational Exposure to a Viral Mimetic Poly(I:C) Results in Long-Lasting Changes in Mitochondrial Function by Leucocytes in the Adult Offspring. Mediators of Inflammation, 2013. 2013: p. 1–8. 10.1155/2013/609602 PMC3793312 24174710

[37] Jao J. and Ciernia A.V., MGEnrichment: A web application for microglia gene list enrichment analysis. PLoS Computational Biology, 2021. 17(11): p. e1009160. 34788279 10.1371/journal.pcbi.1009160 PMC8598070

[38] Langfelder P. and Horvath S., WGCNA: an R package for weighted correlation network analysis. BMC Bioinformatics, 2008. 9(1): p. 559. 19114008 10.1186/1471-2105-9-559 PMC2631488

[39] Langfelder P. and Horvath S., Fast R Functions for Robust Correlations and Hierarchical Clustering. Journal of statistical software, 2012. 46(11). PMC3465711 23050260

[40] Schwartzer J.J., , Maternal immune activation and strain specific interactions in the development of autism-like behaviors in mice. Translational Psychiatry, 2013. 3(3): p. e240–e240. 23481627 10.1038/tp.2013.16 PMC3625915

[41] Ashwood P., , Associations of impaired behaviors with elevated plasma chemokines in autism spectrum disorders. Journal of Neuroimmunology, 2011. 232(1-2): p. 196–199. 21095018 10.1016/j.jneuroim.2010.10.025 PMC3053074

[42] Masi A., , Cytokine levels and associations with symptom severity in male and female children with autism spectrum disorder. Molecular Autism, 2017. 8(1): p. 63. 29214007 10.1186/s13229-017-0176-2 PMC5712192

[43] Arenella M., , Potential role for immune-related genes in autism spectrum disorders: Evidence from genome-wide association meta-analysis of autistic traits. Autism, 2021: p. 136236132110195. 10.1177/13623613211019547 PMC8814945 34344231

[44] Mandal M., , Maternal immune stimulation during pregnancy affects adaptive immunity in offspring to promote development of TH17 cells. Brain, Behavior, and Immunity, 2011. 25(5): p. 863–871. 20854892 10.1016/j.bbi.2010.09.011

[45] Moaaz M., , Th17/Treg cells imbalance and their related cytokines (IL-17, IL-10 and TGF-β) in children with autism spectrum disorder. Journal of Neuroimmunology, 2019. 337: p. 577071. 31671361 10.1016/j.jneuroim.2019.577071

[46] Warren R.P., , Immune abnormalities in patients with autism. Journal of Autism and Developmental Disorders, 1986. 16(2): p. 189–197. 2941410 10.1007/BF01531729

[47] Denney D.R., Frei B.W., and Gaffney G.R., Lymphocyte subsets and interleukin-2 receptors in autistic children. Journal of Autism and Developmental Disorders, 1996. 26(1): p. 87–97. 8819772 10.1007/BF02276236

[48] Han Y.M.Y., , Lymphocyte subset alterations related to executive function deficits and repetitive stereotyped behavior in autism. Research in Autism Spectrum Disorders, 2011. 5(1): p. 486–494.

[49] Weaver C.T., , Th17: An Effector CD4 T Cell Lineage with Regulatory T Cell Ties. Immunity, 2006. 24(6): p. 677–688. 16782025 10.1016/j.immuni.2006.06.002

[50] Filiano A.J., , Unexpected role of interferon-γ in regulating neuronal connectivity and social behaviour. Nature, 2016. 535(7612): p. 425–429. 27409813 10.1038/nature18626 PMC4961620

[51] Nosko A., , T-Bet Enhances Regulatory T Cell Fitness and Directs Control of Th1 Responses in Crescentic GN. Journal of the American Society of Nephrology, 2017. 28(1): p. 185–196. 27297951 10.1681/ASN.2015070820 PMC5198267

[52] Akkaya B., , Tbet provides survival advantage to Tregs during Type 1 inflammation. The Journal of Immunology, 2019. 202(1_Supplement): p. 124.8–124.8.

[53] Doss P.M.I.A., , Male sex chromosomal complement exacerbates the pathogenicity of Th17 cells in a chronic model of central nervous system autoimmunity. Cell Reports, 2021. 34(10): p. 108833. 33691111 10.1016/j.celrep.2021.108833

[54] Ghoreschi K., , Generation of pathogenic TH17 cells in the absence of TGF-β signalling. Nature, 2010. 467(7318): p. 967–971. 20962846 10.1038/nature09447 PMC3108066

[55] Brynskikh A., , Adaptive immunity affects learning behavior in mice. Brain, Behavior, and Immunity, 2008. 22(6): p. 861–869. 18249087 10.1016/j.bbi.2007.12.008

[56] Derecki N.C., , Regulation of learning and memory by meningeal immunity: a key role for IL-4. The Journal of Experimental Medicine, 2010. 207(5): p. 1067–1080. 20439540 10.1084/jem.20091419 PMC2867291

[57] Filiano A.J., Gadani S.P., and Kipnis J., How and why do T cells and their derived cytokines affect the injured and healthy brain? Nature Reviews Neuroscience, 2017. 18(6): p. 375–384. 28446786 10.1038/nrn.2017.39 PMC5823005

[58] Ziv Y., , Immune cells contribute to the maintenance of neurogenesis and spatial learning abilities in adulthood. Nature Neuroscience, 2006. 9(2): p. 268–275. 16415867 10.1038/nn1629

[59] Beumer W., , The immune theory of psychiatric diseases: a key role for activated microglia and circulating monocytes. Journal of Leukocyte Biology, 2012. 92(5): p. 959–975. 22875882 10.1189/jlb.0212100

[60] Koyama R. and Ikegaya Y., Microglia in the pathogenesis of autism spectrum disorders. Neuroscience Research, 2015. 100: p. 1–5. 26116891 10.1016/j.neures.2015.06.005

[61] Courchesne E. and Pierce K., Why the frontal cortex in autism might be talking only to itself: local over-connectivity but long-distance disconnection. Current Opinion in Neurobiology, 2005. 15(2): p. 225–230. 15831407 10.1016/j.conb.2005.03.001

[62] Fuster J.M., Frontal lobe and cognitive development. Journal of Neurocytology, 2002. 31(3-5): p. 373–385. 12815254 10.1023/a:1024190429920

[63] A systematic review of frontal lobe volume in autism spectrum disorder revealing distinct trajectories. 10.31083/j.jin2102057 35364645

[64] Zeestraten E.A., , Sex differences in frontal lobe connectivity in adults with autism spectrum conditions. Translational Psychiatry, 2017. 7(4): p. e1090–e1090. 28398337 10.1038/tp.2017.9 PMC5416715

[65] Constam D.B., , Differential expression of transforming growth factor-beta 1, -beta 2, and -beta 3 by glioblastoma cells, astrocytes, and microglia. The Journal of Immunology, 1992. 148(5): p. 1404–1410. 1538124

[66] Dobolyi A., , The Neuroprotective Functions of Transforming Growth Factor Beta Proteins. International Journal of Molecular Sciences, 2012. 13(7): p. 8219–8258. 22942700 10.3390/ijms13078219 PMC3430231

[67] Meyers E.A. and Kessler J.A., TGF-β Family Signaling in Neural and Neuronal Differentiation, Development, and Function. Cold Spring Harbor Perspectives in Biology, 2017. 9(8): p. a022244. 28130363 10.1101/cshperspect.a022244 PMC5538418

